# Parents’ views of genetic testing and treatment of familial hypercholesterolemia in children: a qualitative study

**DOI:** 10.1007/s12687-018-0373-5

**Published:** 2018-06-14

**Authors:** Karen Forrest Keenan, Robert M. Finnie, William G. Simpson, Lorna McKee, John Dean, Zosia Miedzybrodzka

**Affiliations:** 1 Epidemiology Group, University of Aberdeen, First Floor Health Sciences Building, Foresterhill, Aberdeen, AB25 2ZD UK; 2 Medical Genetics Group, University of Aberdeen, Polwarth Building, Aberdeen, AB25 2ZD UK; 3 Department of Medicine/Care of the Elderly, St. Johns Hospital, Howdon Road West, Livingston, UK; 4 Department of Clinical Biochemistry, Royal Infirmary of Edinburgh, Edinburgh, UK; 5 Department of Clinical Biochemistry, Aberdeen Royal Infirmary, Aberdeen, UK; 6 Health Services Research Unit, University of Aberdeen, Third Floor Health Sciences Building, Foresterhill, Aberdeen, AB25 2ZD UK; 7 Department of Medical Genetics, Ashgrove House, NHS Grampian, Aberdeen, UK

**Keywords:** Familial hypercholesterolemia (FH), Parents, Children, Treatment, Qualitative interviews

## Abstract

Familial hypercholesterolemia (FH) is a serious inherited disorder, which greatly increases individuals’ risk of cardiovascular disease (CVD) in adult life. However, medical treatment and lifestyle adjustments can fully restore life expectancy. Whilst European guidance advises that where there is a known family mutation genetic testing is undertaken in early childhood, the majority of the at-risk population remain untested and undiagnosed. To date, only a small number of studies have explored parents’ and children’s experiences of testing and treatment for FH, and little is known about interactions between health professionals, parents, and children in clinic settings. In this study, in-depth interviews were undertaken with parents who had attended a genetics and/or lipid clinic for FH with their children (*n* = 17). A thematic analysis revealed four main themes: undertaking early prevention, postponing treatment, parental concerns, and the importance of the wider family context. The majority of parents supported genetic testing for FH in childhood. However, although some were very supportive of following early treatment recommendations, others expressed reluctance. Importantly, some parents were concerned that inappropriate information had been shared with their children and wished that more time had been given to discuss how, when, and what to tell in advance. Future research is needed to explore the long-term outcomes for children who undertake genetic testing and early treatment for FH and to trial interventions to improve the engagement, follow-up, and support of children who are at risk, or diagnosed, with this disorder.

## Introduction

Predictive genetic medicine and personalised genomics are heralded as having great potential to improve public health and clinical care, with pre-symptomatic genetic testing expected to become increasingly available for a wider range of common and/or complex disorders (Burton 2011). This form of testing determines whether an individual has inherited a gene, or set of genes, which causes—or increases the risk of—a disease, long before its onset. In choosing to pursue pre-symptomatic genetic testing, patients can use the findings to inform decisions about any early treatment and make behavioural or lifestyle changes to help reduce their risk of developing the disease in later life (Evans et al. 2001; Skirton et al. 2013; Godino et al. 2016). However, studies have shown that there is considerable variation in the uptake of pre-symptomatic genetic testing by at-risk individuals across a range of conditions, even when treatment and/or preventative options are available (Forrest et al. 2012; Nordestgaard et al. 2013; Delikurt et al. 2014).

In this paper, we describe the views and experiences of parents with familial hypercholesterolemia (FH); a serious inherited disorder associated with premature morbidity and death from heart disease. Pre-symptomatic genetic testing is available for individuals at risk of FH, and treatment with medication and lifestyle adjustments has been shown to fully restore life expectancy (Nordestgaard et al. 2013). However, as many as 85% of cases are undiagnosed—and untreated—in the majority of European countries, (Neil et al. 2000; Marks et al. 2004; Nordestgaard et al. 2013), with studies suggesting that there are multiple barriers to cascade screening and treatment of at-risk relatives (Muir et al. 2011; Hardcastle et al. 2015).

FH is an autosomal dominant inherited condition leading to high cholesterol in affected families, where all first degree relatives have a 50% risk of inheriting the condition. It is a “major cause of premature macrovascular arteriosclerotic disease and cardiac death” with 50% of males developing coronary disease by the age of 50 and a 50% mortality by age 60 if untreated (Finnie et al. 2012: 243). Women have a similar spectrum of disease developing 10 years later (Finnie 2010). The commonest cause is a gene change in the LDL (low-density lipoprotein) receptor located on chromosome 19 (Finnie et al. 2012).

FH is considered common, because it is estimated to affect approximately 1 in 500 people in the general population, although recent studies suggest the prevalence is higher (Brice et al. 2013). At the very least, there are between 1.8 and 4.5 million people affected in Europe, with more than 120,000 cases in the UK and over 11,000 in Scotland (Finnie 2010; Brice et al. 2013).

In order to prevent such high rates of disease, a system of cascade screening for heterozygous FH—using genetic testing and family contact methods—was introduced in Scotland in 2008 following UK NICE Guidance (National Institute for Clinical Excellence 2008; Finnie 2010). This system uses index patients who have been diagnosed with FH through genetic testing to alert at risk relatives, in the hope that they too will seek testing and initiate treatment if diagnosed (Finnie 2010; Finnie et al. 2012). The advantage of genetic testing is that it provides index cases with a definitive diagnosis and the aberrant gene can be looked for in relatives, whereas the results of lipid testing may be ambiguous (Hallowell et al. 2016; Personal communication with Dr. Finnie, April 2018).

Family contact methods are a more indirect form of screening, which relies upon family communication, in contrast to direct cascading where a clinic contacts at risk relatives by phone or letter (Hallowell et al. 2011). When using the indirect approach, index patients are asked to pass on personalised clinic letters/information to at risk relatives who have been identified through discussion between a patient and genetic counsellor about family tracing and may benefit from screening (Hallowell et al. 2011). Whilst both methods raise a number of ethical dilemmas, it is acknowledged that the indirect form may result in less uptake and unknowing relatives (Hakimian 2000; Hallowell et al. 2011).

## Familial hypercholesterolemia in children

Studies have shown that initiating statin treatment from childhood can significantly reduce the risk of CVD in adult life (Nordestgaard et al. 2013; Luirink et al. 2015) and that statin use is safe in children (Humphries et al. 2018). As a result, recent European guidance advises that where there is a known family mutation, genetic testing is carried out in early childhood (Nordestgaard et al. 2013). If a positive result is found, children are encouraged to adopt a healthy lifestyle at as early an age as possible; with statin treatment considered by age 10 (Nordestgaard et al. 2013). Thus, whilst genetic testing in childhood for adult-onset conditions is usually not advised (Lucassen et al. 2010), it is considered permissible—even encouraged—in FH where there is “simple” and effective preventative treatment (Nordestgaard et al. 2013; Luirink et al. 2015). This discourse is particularly apparent in the medical literature on FH (Wiegman et al. 2015), as well as in patient organisations where there have been “calls to action” to “bring greater attention to this potentially deadly, but (with proper diagnosis) eminently treatable, condition” (Knowles et al. 2014).

A small body of work has explored parents’ and children’s views about FH, suggesting that there is strong support from families to undertake genetic testing in at risk children, with little evidence of any psychosocial problems (Tonstadt et al. 1995; Tonstad 1996; Umans-Eckenhausen 2002; Jongh et al. 2003). Furthermore, there are limited reports of parents’ declining testing uptake (Weiner 2011), supporting wider studies of favourable parental attitudes towards predictive/presymptomatic genetic testing in children where treatment options are available (Michie et al. 2001). However, one paper does report parental concerns that children may worry about, or resist, their diagnosis (Weiner and Durrington 2008).

In Scotland, the uptake of genetic testing for FH has improved since the introduction of cascade screening (HEART-UK 2013). Nevertheless, the majority of the at-risk population remains untested and undiagnosed, with particular concerns about low uptake rates for children and young people (Familial Hypercholesterolemia Governance Group 2010; HEART-UK 2013). A similar picture has been observed across the UK, where recent studies indicate that cascade screening is underused in children of women with FH (Benson et al. 2016).

Whilst the medical literature now strongly encourages early detection and treatment of FH from childhood, only a small number of studies have explored parents’ and children’s in depth views, and little is known about interactions between health professionals, parents, and their children in clinic settings. The aim of this study was to explore parents’ views and experiences of genetic testing and early treatment of children with FH in Scotland (UK), experiences of their children’s care pathway and to identify any barriers or facilitators in testing and treatment uptake. In-depth interviews with children and young people were also conducted during the fieldwork and will be reported in a separate paper.

## Methods

The data for this paper were generated from a CSO (Chief Scientist Office, Scotland) study, which aimed to explore the “sharing of information between parents and children about genetic risk,” using FH and Huntington’s disease as two contrasting exemplars. The overall study design included four linked phases of research: interviews with genetics professionals; clinic observations; prospective and retrospective interviews with parents and children; and collaborations with patient groups to develop information for children and young people. This paper reports the findings of interviews with parents who have FH.

The study was approved by the North of Scotland Research Ethics Service (REC Ref: 11/AL/0146).

### Inclusion and exclusion criteria

Participants were eligible for the study if they had attended a clinic for FH where cascade testing and/or disclosure to children was raised by the genetics or lipid professional, and they were able to give signed written informed consent.

The clinicians did not invite any participant considered too vulnerable to participate, e.g. those who had had a recent bereavement. Parents whom the clinicians viewed as unable to give informed consent were not included.

### Recruitment and sampling

In the FH group, parents and children were recruited through clinical genetic services and lipid clinics across Scotland between August 2011 and October 2014 by their health professional (a lipid consultant, genetic counsellor or clinical geneticist). The majority of participants were referred for genetic counselling and had attended a lipid clinic. Eleven were recruited by a clinical geneticist, three through a genetic counsellor and three through a lipid doctor. Initially, we planned to recruit from one site (Grampian), but by early 2012, we discovered that only six children had been tested and found to have a gene mutation for FH in that site. At this point, we broadened the recruitment strategy to include two additional sites (Lothian and Greater Glasgow and Clyde).

Potential participants were sent an invitation letter with an information leaflet and a reply slip asking them to contact the researcher if they wanted to participate. All participants had the opportunity to ask questions about the study beforehand and gave written consent. Purposive sampling was used to recruit as diverse a range of participants as possible (Clarke and Braun 2013).

### The participants

In total, 17 interviews were undertaken with parents, 13 had a positive mutation result from DNA testing and a diagnosis of FH, and four were the spouse of a patient with FH. Thus, all parents, apart from four spouses, were mutation carriers and had been interviewed after genetic testing. Three participants had experienced symptoms such as angina or had CVD. All participants had at least one child with an FH mutation. All participants were married, except one who was widowed. All described their ethnicity as “White.” See Table 1 for demographic information.

**Table 1:** Demographic characteristics of participants

Characteristics	*N*	%
Age		
20–29	1	5.9
30–39	2	11.75
40–49	11	64.7
50–59	2	11.75
59–60	1	5.9
Gender		
Male	6	35.3
Female	11	64.7
Education/employment		
Post-secondary qualifications	12	70.6
Full or part-time employment	13	76.5
Homemaker	3	17.6
Retired	1	5.9
Number of children		
One child, with FH mutation	6	35.3
Two children, one with FH mutation	4	23.5
Two children, both with FH mutation	5	29.4
Three children, one with FH mutation	2	11.8

### Data collection and analysis

Semi-structured interviews were conducted (by KFK) with participants and lasted between 30 and 90 min. The majority of interviews took place in participants’ homes or a hospital clinic; two were by phone. All phone and in-person interviews were audio-taped with consent and fully transcribed. Promises of anonymity and confidentiality were given. The interviews focussed on: family history of FH, reasons for seeking childhood testing, parents’ experiences of the testing process, views of testing and treatment in childhood, the impact of any result, and any gaps in information or support. Clinic observations were also undertaken to enable the researcher to learn more about patient care for FH.

The analysis was conducted in two main phases. An initial thematic analysis was undertaken using a system of open coding, the constant comparison method, and a search for negative cases (Strauss and Corbin 1990; Guest et al. 2011). KFK identified themes and categories by reading and re-reading transcripts. Different segments of the data were then grouped together into separate categories, allowing reflection on the major themes and any relationships among them. The thematic analysis was ongoing throughout the fieldwork until a point of data saturation was reached about the main themes (Strauss and Corbin 1990). In the later phase of analysis, the findings were interpreted with reference to existing psychological and social theory on families affected by inherited conditions, where issues related to engagement with genetic risk (McAllister 2003; Frich et al. 2007b), discourses of genetic risk and responsibility (Hallowell 1999), patient (dis)empowerment (McAllister et al. 2011), illness experiences (Frich et al. 2007a; Mackie et al. 2015), and personal theories of inheritance (Wilson et al. 2004; Frich 2006) were anticipated to arise. We also anticipated that family communication about genetics would influence parental attitudes and behaviour in this area (Forrest et al. 2003; Wilson et al. 2004; van den Nieuwenhoff et al. 2007). Thus, whilst phase one aimed to generate inductive themes from participants’ accounts, phase two interpreted these accounts within a broader theoretical framework.

## Results

We identified four main themes from parents’ interviews about their experiences of genetic testing and treatment for FH in their children. These were the following: (1) undertaking early prevention; (2) postponing treatment; (3) parental concerns; and (4) the wider family/social context. Within each main theme, we identified several subthemes (see Table 2).

**Table 2:** Main themes and subthemes

Themes	Subthemes
Undertaking early prevention	Protecting children’s future health; awareness of heart disease in family from childhood; trust in health professionals; telling children about FH
Postponing treatment in children	Parental responsibility to assess risks versus benefits; differences in medical advice; gender; low estimation of risk
Parental concerns	Age inappropriate communication with children in clinic; giving blood in childhood; statin treatment in children; normalisation over medicalisation
The wider family context	Parenting threatened; making family comparisons.

### Undertaking early prevention

Early prevention was a prominent theme, with parents citing their support for statin treatment and lifestyle adjustments in childhood. Parents described several key factors, which influenced their decision, such as witnessing serious FH events in other relatives, experiencing their own symptoms, knowing there was a family history of heart disease from an early age, and having trust in their health professional. The need to tell children about their risk, or diagnosis of FH, was threaded throughout these accounts.

#### Protecting children’s future health

Just over half of participants supported the genetic testing of children for FH *and* instigating statin treatment from an early age, asserting that preventative medication would protect their child’s future health. A consistent theme was that participants had witnessed serious FH related events in other close relatives or had experienced symptoms themselves. These experiences created a strong desire to “look after” their children’s future health, with statin treatment viewed as a key mechanism of prevention. For example:

We want to help him, give him as good a shot as he can get … When you look at what happened to my dad - at 47 he had a heart attack … You just think it’s your son! You want to wrap them up in cotton wool … [So] we’ve decided to give him statins until he’s 16 … We’ve covered him until he’s old enough to decide for himself. (FH7)

If that’s what it takes then they’ll get them … I mean my daughter lost her sibling, you take note of that … and look after your family. It was so obvious that it was coming down the line. (FH1)

#### Awareness of heart disease in family from childhood

Several parents reported being aware of a family history of heart disease during their own childhood and that they had always felt at high risk themselves. As a consequence, they had taken preventative measures throughout their lives and were supportive of genetic testing and treatment for FH in their children. However, whilst one participant grew up knowing about FH specifically—and had taken statins since childhood, the other had had little knowledge of the condition itself, or the prescription of statin treatment for FH, until recently. Although he had followed a “strict” regime of diet and exercise, describing a “very clean” lifestyle, he still developed angina in his 30s and asserted that recent developments in research would help his children:

It’s more than 20 years ago since my father died. So it was in my mind all the time that there is a sign, especially with my uncle dying too … In my case it seemed to be learning things a little bit too late [about FH] because I didn’t take any statin or anything. It became clear 2–3 years ago, even though I live a healthy lifestyle, the faulty gene is the root of my problem and if I don’t have an operation or do something I will probably die …. I would have been taking statins before but because of what I don’t know [I didn’t]… That's why research and everything it would help my children. (FH10)

In contrast, this mother described how:

I’ve known about FH since I was a kid and have always been on some kind of medication … My dad’s now in his 70s … It’s not something I feel particularly threatened about having … People on statins live a long long time … So I was absolutely keen to get my son tested. (FH16)

#### Trust in health professionals

Participants also reported having a sense of trust in their doctor, or genetic counsellor, particularly if they had a long-term consistent relationship with the same health professional *and* their cholesterol levels had been lowered following advice to take statins. If parents trusted their health professional then they were more likely to be receptive about advice to seek genetic testing and statin treatment in their children. For example:

I think with Dr X, because we’ve been working together for that long, and I’ve seen my cholesterol come down, I would totally trust his judgment on what's the right thing. Whatever he's saying I would accept really. (FH18)

#### Telling children about FH

In this theme, parents also supported telling children about FH, with some encouraging openness from a young age, asserting that this would help children adjust to any diet, medication, and lifestyle changes.

It’s better to know now from an early age and to adjust to that straightaway … If you can get them when they’re young and impressionable that becomes a habit. (FH23)

However, others indicated that they had taken, or would take, a more incremental approach to telling their children, which was dependent on their child’s age, medical advice, and whether there was any action that could be taken or not. For example:

I wouldn’t want him to find out too young. I’ll probably wait until he’s a bit more mature then sit down and explain it to him. He could get worried. I don’t think it’s anything to be worried about, but you never know how he’ll feel and how he’ll react to it. (FH7)

Obviously when children are quite young you can’t give them information that they can’t do anything with, other than worry. I did try and do it … according to their age, and what needed to be done as I was told at the time … and the doctor said there’s no point doing anything until after puberty. (FH5)

In this case, a participant had sought advice about her daughter’s risk of FH from her GP, but was advised that there wasn’t anything that could be “done” until after she finished puberty. Thus, whilst she told her daughter about her risk of FH when she was in her early teens, they postponed “doing anything” until adulthood, intimating that this included fully “educating” her about the family history. As a consequence, she reflected that:

My daughter can’t understand why I didn’t give her more details when she was younger … but things have come on so quickly. (FH5)

In other families, participants reported a much more limited approach to disclosure, deferring responsibility for educating their children about FH to a health professional:

I’ve never really spoken with her myself about FH, but the genetic counsellor told her all about it. We actually don’t talk about it, do you know what I mean? (FH 28)

Some participants also asserted that they used health professionals in a supporting role to inform their children about FH:

It’s reinforcement for the children, it was better coming from professionals than from us – saying this is what you should be eating. (FH3)

### Postponing treatment in children

In this theme, less prominence was given to medical authority, with parents asserting that they had a responsibility to assess the risks versus benefits of early treatment themselves. A few participants had received differing advice from health professionals about the need to initiate early statin treatment in children. We also found participants who felt they had a low risk of developing CVD, citing gender as a protective factor.

#### Parental responsibility to assess risks versus benefits

Several participants questioned the benefits of initiating statin treatment in children. Whilst these parents did express trust in their health professional, it was countered with a sense of parental responsibility to question the advice they were given, particularly if they had previous experience of poor health care or lack of follow up. Parents described taking “ownership” of their children’s healthcare by seeking further information about FH and probing their health professional about risks versus benefits.

Although I trust the doctors I know that having seen the treatment my mum got, I'm not saying that she bad treatment … but there was not much in the way of follow up. So you can’t always assume that it’s going to come to you and somebody is going to come and give you an answer … I think you've got to try and think about it yourself as well, and take ownership of it, not see it as somebody else’s responsibility … So I might push it in terms of trying to find out what the benefits are. (FH23)

#### Differences in medical advice

Participants also reported receiving conflicting advice from different healthcare professionals (e.g. their GP, paediatrician, lipid consultant, or genetic counsellor) about the importance of genetic testing and statin treatment in children for FH. Whilst a sense of “trust” in a medical doctor was again evident, participants were uncertain about the benefits of early statin treatment, questioning why it should be initiated so young, particularly if other health professionals were confident about delaying. For example:

I think we had two slightly different views, the genetic counsellor was more relaxed about it, the medical doctor less so. And it’s hard then to know which to go with … At the end of the day I think I trust the doctors … But I might ask questions to have a better understanding of the advice they’re giving. So for my daughter, I would ideally like to postpone her taking statins for as long as possible. (FH23)

Others highlighted that medical knowledge and treatment advice about FH has changed over time, which could also reinforce uncertainty about early statin treatment being the “right solution” for children.

We were told there was no point in testing children until they were teenagers and it wasn’t such a problem for females, it was a male thing. But I think we’ve all come past that now. (FH2)

As to the ideal treatment … I don’t think the medical profession could guarantee 100% that they have the right solution, the right solution changes on what decade you’re in I think. (FH27)

#### Gender

A few participants asserted that gender could be a factor in postponing statin treatment in female children. Currently, medical advice is to stop taking statins in pregnancy and when breastfeeding; given this advice, it was envisaged that teenage girls and young adult women may choose to postpone any statin treatment until their 20s—or even their 30s. For example,

I think any female receiving that information is going to question whether she needs to take the drug until after … she’s had her family. (FH14)

The same participant also admitted that she had chosen to delay genetic testing and statin treatment because she was premenopausal, asserting that this gave her some “protection” against heart disease.

I assumed that … because of my age … I was protected. (FH14)

#### Low estimation of risk in children

In some accounts, it was clear that FH was not viewed as an overly threatening condition by a parent nor to the health of their children. In these circumstances, there was no sense of urgency about children starting statin treatment, which could extend to adult children too, particularly if a parent had not experienced any symptoms or anxiety about FH themselves.

I mean this is high cholesterol, I know it has its problems, but it’s not a scary one. I haven’t got any fear that my heart is about to stop. (FH14)

This parent was unconcerned when her adult daughter also chose to delay treatment, commenting that:

Her cholesterol is six and she just says ‘look I’ll deal with it!’ [Laughter]. So I presume she will one day. (FH14)

### Parental concerns

Throughout these accounts, parents voiced a number of concerns, primarily about when to initiate treatment for FH in one’s children and how best to manage their clinical care. Two further concerns related to parents’ experiences of inappropriate communication with their children in clinics and a desire to retain normality over medicalisation, with some highlighting the dilemmas of genetic testing in childhood.

#### Inappropriate communication with children in clinic

Several participants reported concerns about the information that professionals had given to their children at different ages/developmental stages during consultations about FH, for example, contraceptive advice given to a 9 year old by a doctor, which was felt to be highly inappropriate.

The doctor at the hospital went into far too much depth. He was talking about contraception and things. I’m sitting going she’s only nine! She actually doesn’t know what sex is. A lot of it just went totally over her head. (FH28)

In contrast, this mother and her daughter had also been to a consultation with a genetic counsellor who:

Just totally talked to her, more than talked to me. Totally down to her level, explained it real good, didn’t explain anything long. You can give too much information that’s not needed. (FH28)

One couple also reported concerns about *how* information about FH had been conveyed to their child during a conversation at a family appointment when they had raised questions about the benefits of early treatment, describing a lack of parental consultation or involvement in this process.

Father: The doctor was clearly quite anxious that he laid the facts very bare before us, I think to let us understand the importance of accepting the drugs … And perhaps there was more attention paid *to that aspect of it* than there was to the effects information like that could have on the mind of a 13 year old. He put it very very starkly … I’m not saying that that information should not have been shared with us, but it should probably have been shared *with us*, privately, rather than with all three of us, to allow us to judge whether or not it was something we would want shared with our daughter at that stage. (FH29)

Mother: So I think for me a separate appointment would have been helpful for us to discuss our concerns, and then have had the joint appointment with our daughter as a follow-up … We’re saying the lipid specialist should have better training in communicating with children. That was clearly missing.

There was also concern from parents about how medical professionals would frame future conversations with their children, particularly around healthy eating, with some mothers asserting anxiety about the possibility of eating disorders in teenage girls, and how to balance this with information about FH.

I’m very conscious of wanting my children to have a very healthy relaxed relationship with food. I don’t want her to have that ‘fat is bad’ message… I don’t want that from a consultant … Screwing with a teenagers relationship with food is a volatile playground … [You need to] know what age person you’re talking to and what the other vulnerabilities are. (FH26)

#### Giving blood in childhood

Having to give blood in childhood was also raised as an important concern by parents as it was usually the first time that children had experienced venepuncture. Whilst it was intimated that this may not be a huge concern for adults, there was an appeal to view this from a child’s perspective. Thus, according to one father:

The thing that has absolutely traumatized her is going to give blood … She just reacted really badly to it. (FH18)

Unfortunately, the experience of giving blood was very distressing for this participant’s daughter, which he felt had been intensified by attending an adult lipid clinic with no specific facilities or advice for children, e.g. to apply anaesthetic cream beforehand.

#### Strong concerns about statin treatment in children

Three parents reported having strong concerns about statin treatment in children, not only because of their long-term safety but also potential side effects. They had all sought further information from the medical literature—and from other patients—and concluded that the “evidence base” was equivocal; one queried whether statins were in fact “dealing with the right problem.” For example:

It is a recent drug, and you don’t know what the long term effect could be. And I’ve spoken to so many people that have said they have this muscle ache … So how’s my child going to know when to say ‘I don’t actually feel quite right mummy?’ So I wouldn’t be keen to put a child on a tablet, especially if he’s fit and healthy. (FH8)

I have concerns about what it does and doesn’t mean and if statins are the right approach when it comes to my daughter … It’s one thing when someone of 50 or 60 goes on to it, we’ve got a lot of experience with people at that age. But the idea of putting something into somebody who is not yet fully grown, that we don’t even really know for sure is dealing with the right problem freaks me out. (FH26)

These participants also reported a lack of trust in the medical profession, citing historical examples of medical mistakes, which have resulted in serious harm to children.

You don’t ever like the idea of your child being any sort of a guinea pig, even if it's done … with the best of intentions … I just think Oh God! … My husband is from the Thalidomide era you know! (FH26)

#### Normalisation versus medicalisation

Whilst the primary concern of these parents was to avoid any long-term harms from statin treatment in childhood, they also wanted to protect the *current* health and wellbeing of their children, indicating a desire to maintain normality and protect “childhood” itself from medicalisation. For example:

The reality of it when it sinks in that your daughter has a genetic condition, it’s hard on a number of levels … It’s hard because you don’t want that for any of them … As a parent you want your child to live their childhood carefree, happy, skipping through the wind! You don’t want them burdened with this. (FH26)

Protecting children from medical intervention could also extend to notions of heredity and genetic risk. For example, in a joint interview with a mother and son (aged eight), his mother spoke in whispers when using words, such as “hereditary” or “genetic.”

In some cases, parents made the decision that their children were “far too young” to begin lifelong treatment on statins and chose to disengage from the paediatric and genetics service, asserting that they would return when their children were old enough “to decide for themselves.” One parent had not attended any further appointments with her subsequent children, commenting that:

Part of me thinks I should never have got involved with this. (FH8)

Three parents described their feelings about the dilemmas of genetic testing and the consequences this may have for their children, acknowledging that whilst there may well be long term health benefits, there can also be negative implications:

I feel a bit damned if you do, damned if you don’t. It’s the challenge with genetic testing – on the one hand its clearly better to know, on the other it does bring its own set of worries. She’s not going to have as many options as someone without those concerns would have. (FH26)

### Wider family context

Another key theme in parental accounts was the importance of the wider family context in relation to views about genetic testing and treatment of children for FH. Testing for FH may be particularly sensitive if parenting itself had been previously threatened, or was felt to be under threat. Participants also compared family members to each other to assess their own personal or child’s risk, with some extending this beyond FH.

#### Parenting threatened

In a few cases, parents’ accounts revealed sensitive circumstances where they had had to protect their children and/or where parenting itself had recently been threatened, e.g. parental divorce or a parent’s ill-health, which appeared to heighten their anxiety about their children’s welfare and feelings of protectiveness about their children’s health. For example, one mother described the difficult time she had during her pregnancy:

I ended up spending most of the pregnancy absolutely terrified that I was doing something to her … I had to go through drugs [in my pregnancy] that quote unquote they ‘did not know to be unsafe’. That’s not the same thing as safe! … I don’t make any connection between that and the FH, but it’s like … oh look, we’ve finally got this baby! (FH26)

Throughout her interview, this mother was clearly anxious about the potential of FH treatment “doing something” harmful to her child.

We also observed a parent being offered a referral to “psychology” to discuss the concerns she had about testing and statin treatment in her children, and to help with her “decision making,” from which she left visibly upset (Fieldnotes from clinic observation, 2013). However, during her study interview several months earlier she had already asserted her view that:

While they are young and they are little, for me, it’s a no. And if that decision were taken away from me then I would be quite upset about it. (FH8)

#### Making family comparisons

Similar to other participants, one mother compared herself with her siblings who had high cholesterol—assessing that their risk of developing heart disease was far higher than her own because of lifestyle factors such as smoking, excessive alcohol intake and poor diet. As a result she deduced that her risk of heart disease—and her children’s—was lower than her relatives, insinuating that luck and chance also play a role:

My brother just had a heart attack … but he’s a heavy smoker, doesn’t eat well and is overweight. I mean I’m not a particularly great eater, but I don’t smoke and I don’t drink excessively, and then you know there are people who are perfectly healthy [and have a heart attack]! (FH8)

Parents also described making health comparisons between their children, as well as examples of children comparing their health with that of siblings, leading one mother to assert:

Strangely she’s the one that’s inherited all the ailments – she’s the one that has to wear glasses, she’s the one that got asthma and is also the one that’s got high cholesterol … In her younger days she asked why am I the one that has all the problems? (FH2)

A few participants also described other serious illnesses they had witnessed within their family, for example, breast or ovarian cancer, acknowledging that they would be supportive of pre-symptomatic genetic testing for other conditions if it was available for their children.

What I would really like to say to them is what else can you test her for? Let’s test her for the genes that predict anything else. (FH27)

## Discussion

This article is the first to explore UK parents’ in depth views and experiences of genetic testing and treatment for FH in children. A qualitative analysis was undertaken, which identified four main themes from parents’ accounts: (1) undertaking early prevention; (2) postponing treatment; (3) parental concerns; and (4) the wider family context.

A key finding was that just over half of parents (*n* = 9) were supportive of genetic testing for FH in children and early treatment with statins. This is similar to previous studies of parental attitudes towards genetic testing and treatment of FH in children, which found that the majority of parents had a positive attitude towards screening children for FH (Tonstadt et al. 1995; Tonstad 1996; Umans-Eckenhausen 2002). Also similar to previous studies, we found that many participants had a strong sense of illness vulnerability from experiencing personal symptoms themselves or witnessing serious illness events in close family members (Frich 2006; Jenkins et al. 2012), creating a desire to protect their children’s health and avoid repeating history. Our findings support studies of genetic responsibility where parents feel they are “doing the right thing” by seeking genetic testing and undertaking risk management strategies, particularly if they are a new parent (Hallowell 1999; Frich 2006). Indeed, one of the main benefits of DNA testing for FH maybe to provide information for future generations (Hallowell et al. 2016).

We also found participants who had grown up with a well parent whom they had witnessed undertaking long-term preventative measures and was viewed as a role model for FH related health behaviour, which was now encouraged in their own children. Our findings support other work, which suggests that these participants were “intensely engaged” with their risk (McAllister 2003) and felt a shared sense of “family identity” (Jenkins et al. 2012), highlighting the importance of prior family experiences (Mackie et al. 2015). However, FH was not viewed as a significant burden, but more of a lifestyle adjustment, involving a healthy diet, exercise, and statin treatment from an early age. This could be viewed as an example of preventative genetic medicine occurring “down the line” through several generations, similar to clinic observations in other serious inherited conditions. For example, hereditary breast/ovarian cancer where several generations may choose to undertake a preventative mastectomy (personal communication with ZM). Having a strong sense of trust in the medical profession as a whole and a long-term consistent relationship with an FH health professional was also an important factor in testing uptake and acceptance of treatment in participants’ children.

In contrast to other studies, an important finding was that a subgroup of parents were more reluctant about early statin treatment in their children, preferring to delay this for as long as possible. Participants with poorer experiences of healthcare and those who were active information seekers tended to challenge professional advice, asserting that it was a parental responsibility to assess any risks versus benefits of early treatment with medication. Our findings also draw attention to participants who had received conflicting advice from different healthcare professionals about the importance of early statin treatment in children, raising dilemmas for parents about whose advice to follow. This supports other studies, which suggest that uptake rates for FH differ according to the healthcare setting patients attend, e.g. general practice versus lipid clinics (Jenkins et al. 2012). Given that there are differences in professional views about the ethics and implementation of preventative genetics in children, including FH, this may influence the advice which parents receive (Chapple et al. 1996; Will et al. 2010).

Another important finding was that participants with a less visible family history of FH, who had no contact with affected or at-risk relatives, or for whom FH was a recent diagnosis, had little sense of an FH “family” or “genetic” identity, and perceived the condition as less threatening (Frich 2006; Weiner and Durrington 2008; Claassen et al. 2010; Jenkins et al. 2012). As a result, there was less incentive to seek genetic testing in children, or comply with early treatment advice, with a similar pattern of procrastination observed in participants’ adult children. Gender was also cited as important in young women’s decisions to delay or use treatment intermittently as women may not want to initiate statin treatment until after they have completed their family, and being premenopausal was viewed as a “protective” factor against risk of heart disease. Similar to previous studies, parents also drew on more environmental models of the “coronary candidate” and notions of luck and chance, which lessened their personal sense of vulnerability—and risk to children (Frich et al. 2007b; Weiner 2009). In addition, we observed parents who compared themselves with other high risk family members, calculating their own risk as lower (Frich 2006).

In contrast to previous studies, our findings draw attention to a subgroup of parents who were particularly anxious about initiating early statin treatment in their children, questioning the safety of using statins in children and the lack of long-term empirical evidence about their efficacy. Parental reluctance, or anxiety, about the use of statins in childhood exemplifies the ambivalence, fear, and responsibility, which many parents expressed about the management of their children’s health, particularly in relation to early treatment (von der Lippe et al. 2017). In addition, our study lends some support to previous work in other adult-onset inherited conditions, which found that parents may place a high value on maintaining “normality” (Forrest Keenan et al. 2015) and protecting childhood from potentially unnecessary medical intervention and surveillance.

Similar to other studies, our data also draws attention to the importance of a parent’s psychosocial context, as genetic testing in children may bring sensitive personal and family issues to the surface, e.g. guilt and shame (Wilson et al. 2004; Frich et al. 2007a), or even threaten parenting itself. Our findings support the work of McAllister (2003:500) who suggests that when participants experience engagement as “too painful,” they may disengage, even if they were previously “intensely engaged” with their risk. In these circumstances, we observed that parents may disengage their children, or choose not to refer any subsequent children. In addition, our findings draw attention to parents who felt pressured to initiate early statin treatment in their children, reinforced by very graphic information, or even a psychology referral to “help with decision-making” which may ultimately disempower, rather than empower, patients with inherited conditions (McAllister et al. 2011; Metcalfe et al. 2011). Similar to our previous work, we observed that parents may also be “coming to terms” with their own, and/or their children’s diagnosis, and were either “not ready” to comply with treatment advice or used distancing as a coping strategy (Forrest et al. 2003; Frich 2006; van den Nieuwenhoff et al. 2007). Taken together, these parental concerns may go some way to explaining barriers to uptake and initiation of early treatment. Our findings also raise questions about the long-term implications for children known to have FH if parents choose to disengage them from FH services, although the use of a more direct contact, or a mixed approach, to cascade screening could help address this issue (Newson and Humphries 2005; Hallowell et al. 2011).

Whilst disclosure to children about FH was not generally portrayed as a burden by participants, our findings draw attention to the supportive role of health professionals in sharing genetic risk information with children (Forrest et al. 2003; Metcalfe et al. 2011). Similar to other studies, we found parents who only “alerted” children about their risk of FH, but deferred responsibility for fully “educating” them to a health professional (van den Nieuwenhoff et al. 2007), although disclosure could also be a more joint and shared process between parents and health professionals (Cribb and Entwistle 2011). Nevertheless, a new finding is that children and parents had experienced “unsanctioned” disclosure, where health professionals had presented “stark” or inappropriate information, with little or no clinical time given to seeking parental views about the process of disclosure itself. Given the importance of parental “readiness,” and the potential to disengage, this seems an important but potentially neglected aspect of care which is likely to influence a parent’s sense of empowerment and engagement.

## Limitations of study

There are several limitations to our study. Firstly, it is a UK study and the data may not apply to other populations, where there are different screening methods and healthcare systems. A second limitation is the small sample size, although we did broaden our recruitment strategy to include different geographical locations, increasing the diversity and range of experiences. A further limitation is that we recruited more female than male participants. Our findings may also be subject to recruitment bias as patients who are more supportive of testing and treatment in children may be more likely to participate in research. Future studies would benefit from recruiting more fathers, exploring the views of children and young people, and those who have declined testing.

## Conclusion

This paper adds to a growing body of work about preventative genetics and the role of parents as gatekeepers in childhood genetic testing for serious treatable inherited conditions like FH. Whilst just over half of parents supported genetic testing and early treatment recommendations in children, others expressed several concerns, particularly about the use of statins in childhood. Parental engagement was influenced by having a strong sense of personal vulnerability, open styles of family communication, and trust in the medical profession. Disengagement of children occurred when parenting was threatened and patients felt disempowered. In general, parents did not feel that disclosure to children about FH was a burden. However, some were concerned that inappropriate information had been shared with their children and wished that separate time had been given to discuss how, when, and what to tell them. Future research is needed to explore the long-term outcomes for children who undertake genetic testing and early treatment for FH and to trial interventions to improve the engagement, follow-up, and support of children who are at risk, or diagnosed, with this disorder.
